# Predictors of loneliness onset and maintenance in European older adults during the COVID-19 pandemic

**DOI:** 10.3389/fpsyg.2023.1172552

**Published:** 2023-06-02

**Authors:** Vania Panes Lundmark, Maria Josefsson, Anna Rieckmann

**Affiliations:** ^1^Umeå Center for Functional Brain Imaging, Umeå University, Umeå, Sweden; ^2^Department of Integrative Medical Biology, Umeå University, Umeå, Sweden; ^3^Department of Statistics, Umeå School of Business, Economics, and Statistics (USBE), Umeå University, Umeå, Sweden; ^4^Centre for Demographic and Ageing Research (CEDAR), Umeå University, Umeå, Sweden; ^5^Institut für Psychologie, Universität der Bundeswehr München, Neubiberg, Germany

**Keywords:** longitudinal, predictors, loneliness, transient loneliness, persistent loneliness, chronic loneliness, COVID-19, pandemic

## Abstract

**Objectives:**

Loneliness is a major public health concern. Duration of loneliness is associated with severity of health outcomes, and further research is needed to direct interventions and social policy. This study aimed to identify predictors of the onset vs. the maintenance of loneliness in older adults before and during the pandemic using longitudinal data from the Survey of Health, Age, and Retirement in Europe (SHARE).

**Methods:**

Groupings of persistent, situational, and no loneliness were based on self-reports from an ordinary pre-pandemic SHARE wave and a peri-pandemic telephone interview. Predictors were identified and compared in three hierarchical binary regression analyses, with independent variables added in blocks of geographic region, demographics, pre-pandemic social network, pre-pandemic health, pandemic-related individual, and country level variables.

**Results:**

Self-reported loneliness levels for the persistent, situational, and no loneliness groups were stable and distinct through 7 years preceding the pre-pandemic baseline measure. Shared predictors were chronic diseases, female sex, depression, and no cohabitant partner. Persistent loneliness was uniquely predicted by low network satisfaction (OR: 2.04), functional limitations (OR: 1.40), and a longer country-level isolation period for older adults (OR: 1.24).

**Conclusion:**

Interventions may target persons with depression, functional limitations, chronic health issues, and no cohabitant partner. The added burden of the length of isolation on those who are already lonely should be taken into account when employing social policies that target older adults. Further research should distinguish between situational and persistent loneliness, and seek to identify predictors of chronic loneliness onset.

## 1. Introduction

As lockdowns and social distancing measures were employed in order to contain the spread of COVID-19, the alarm of a potential “loneliness pandemic” was raised (Armitage and Nellums, 2020; Killgore et al., 2020; Wu, 2020; Laranjeira, 2021). Pandemic-related restrictions have a direct effect on social isolation, defined as the *objectively measured* absence of social contacts, relationships, or activities (e.g., Davies et al., 2021). Loneliness is a related but distinct construct referring to the *subjective experience* stemming from the actual or perceived absence of social relationships that serve to meet basic emotional needs (Park et al., 2020; Quadt et al., 2020). While social isolation and loneliness do not necessarily co-occur (Park et al., 2020), a recent meta-analysis of 24 longitudinal studies found a small but significant increase in loneliness since before the outbreak of COVID-19 (Ernst et al., 2022). Loneliness is a unique risk factor for heightened mortality risk (Holt-Lunstad et al., 2015; Rico-Uribe et al., 2018) and is associated with a number of adverse health outcomes including depression (Quadt et al., 2020), cardiovascular disease (Lim et al., 2020; Park et al., 2020), and dementia (Rawtaer et al., 2017; Lara et al., 2019; Lee et al., 2022; Salinas et al., 2022). The older adult population, being simultaneously vulnerable to COVID-19 infection, subjective loneliness, and cognitive decline, has been identified as a particular risk group (Lara et al., 2019; Luchetti et al., 2020; Atzendorf and Gruber, 2021; Piolatto et al., 2022).

Identification of concomitants and modifiable risk factors of loneliness is an important step toward developing effective interventions targeting relevant risk groups. In cross-sectional and longitudinal research, a large number of variables have been identified as correlates or risk factors of loneliness. Among adults over 50 years of age, higher prevalence of loneliness has been found with advancing age (Yang and Victor, 2011). Lower education level, migrant status, and living in a nursing home or a rural rather than an urban area have also been associated with higher levels of loneliness (Cohen-Mansfield et al., 2016; Vozikaki et al., 2018; Lim et al., 2020), and a larger incidence of loneliness has been found among individuals suffering from physical and mental conditions (Cacioppo and Hawkley, 2009; Kim et al., 2020; Park et al., 2020), including alcohol abuse (Åkerlind and Hörnquist, 1992). While loneliness is a known risk factor for, or prodrome of, cognitive impairment (Rawtaer et al., 2017; Lara et al., 2019; Lee et al., 2022; Salinas et al., 2022), cognitive decline may simultaneously increase the risk of social isolation and loneliness over time (Hackett et al., 2019). So far, cognitive impairment has been understudied as a risk factor for loneliness (Dahlberg et al., 2022).

Despite the large number of correlates and risk factors identified, a recent review of longitudinal studies by Dahlberg et al. (2022) found that only a few risk factors have been consistently related to loneliness in multivariate analyses in older adult samples, describing the evidence base as “broad but shallow.” Consistent associations were found for not being married or partnered, having a limited social network, low social activity level, poor self-rated health, and depression. While representing distinct entities, depression and loneliness often co-occur and may exacerbate each other over time (Luanaigh and Lawlor, 2008). Dahlberg et al. (2022) found that female sex was only significant in bivariate analyses, and concluded that this relationship may be mediated by other factors more commonly seen in females, such as widowhood.

Crucially, loneliness has been characterized as equally state–and trait-like (Mund et al., 2020a,b). The short-term, state-dependent, dynamic component of loneliness has been termed situational or transient loneliness, while the temporally stable trait component has been referred to as chronic or persistent loneliness. To date, clear definitions and distinctions between the terms situational and transient, as well as chronic and persistent, are lacking, and we will therefore use them interchangeably. The differentiation of persistent from transient loneliness is important because chronic loneliness is associated with worse health outcomes compared to short-term loneliness (Cacioppo and Hawkley, 2009; Cacioppo and Cacioppo, 2018; Martín-María et al., 2020). For older adults, persistent loneliness is associated with greater cognitive decline (Zhong et al., 2016), higher risk of dementia onset (Akhter-Khan et al., 2021), more frailty (Chu and Zhang, 2022), and higher mortality rates (Shiovitz-Ezra and Ayalon, 2010). This is in line with conceptual models portraying loneliness as a complex biopsychosocial process (Qualter et al., 2015; Cacioppo and Cacioppo, 2018). While state loneliness is common and may be adaptive by preventing damage to the “social body,” these models link duration of loneliness to severity of health outcomes through “wear and tear” by hypervigilance to social threats, avoidance, and maladaptive thought patterns leading to an allostatic overload (Quadt et al., 2020). In 2022, the Tackling Loneliness Evidence Group of the United Kingdom Department for Digital, Culture, Media and Sport (DCMS) published a report stating the need for a clear distinction between transient and chronic loneliness (McDaid et al., 2022), highlighting the need for further research into whether the predictors of transient and chronic loneliness are shared or distinct. Although one paper (Yang, 2018) reported largely similar risk factors for situational and persistent loneliness, research on this topic has been sparse.

While studies of general loneliness in the COVID-19 pandemic have not identified any pandemic-specific risk or protective factors (Buecker and Horstmann, 2021), they do not control for the fact that a portion of participants likely experienced chronic loneliness already before the pandemic. In addition, studying the effects of country-level pandemic-related factors, such as social policies, requires large surveys with cross-country data, perhaps explaining why this has not been widely done. In one paper by Atzendorf and Gruber (2021), self-rated increase of depression and sadness, but not loneliness, was more common in countries with a higher number of COVID-19 related deaths per million inhabitants, as well as more stringent socially restrictive measures.

To date, many studies have focused on general levels of loneliness in response to the COVID-19 pandemic, as reviewed by Buecker and Horstmann (2021), Ernst et al. (2022), and Lazzari and Rabottini (2022). We extend this research by focusing on duration of loneliness, distinguishing between the risk and protective factors for developing, or maintaining, feelings of loneliness. We hypothesize that loneliness measured during the pandemic captures both recent onset (situational) and long-term (persistent) loneliness, and that there are distinct predictors for loneliness onset and maintenance. Using longitudinal interview data from the cross-national Survey of Health, Aging and Retirement in Europe (Börsch-Supan et al., 2013), we aim to identify pre-pandemic and pandemic-specific predictors of peri-pandemic loneliness onset and maintenance in older adults. We account for the simultaneous measurement of both state and trait loneliness by dividing the sample into groups based on pre-pandemic loneliness levels. Using weighted hierarchical logistic regression analyses, we investigate effects of commonly studied factors added in blocks. The design allows us to evaluate unique first order effects of each predictor candidate, while controlling for other potentially influential factors including geographic region and residential area, age, sex, cohabitation, widowhood, education, cognitive and mental health, social isolation, and network satisfaction. We also include national and individual pandemic-specific measures, such as COVID-19 deaths per million inhabitants, length of recommended or required self-isolation period for older adults, frequency of personal contact with others, and leaving home since the start of the pandemic.

## 2. Materials and methods

### 2.1. Sample

**FIGURE 1:**
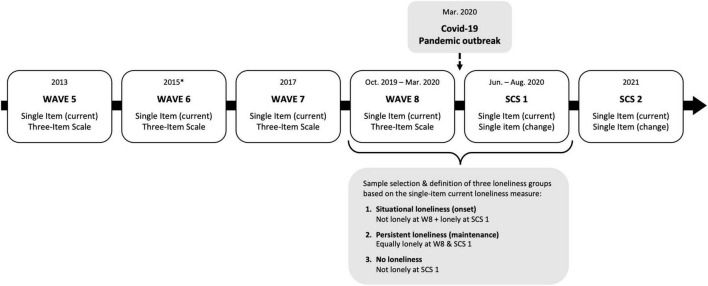
Timeline with overview of time points, available loneliness measures, and definition of loneliness groups used in the study. Three assessments of loneliness are available: 1. A single-item question asking about current loneliness, 2. A three item loneliness rating scale, 3. A single-item retrospective change question. Loneliness groupings were derived from the single-item current loneliness because it was asked at every wave and the SCSs. *Wave 6 data collection in Netherlands ended in 2016.

The sample comprised older adults from 26 European countries and Israel, participating in SHARE before and during the COVID-19 pandemic. The sample was limited to participants taking part in the 8th regular SHARE wave interview, between October 2019 and March 2020 (Bergmann and Börsch-Supan, 2021; Börsch-Supan, 2022g), and a peri-pandemic telephone interview (SHARE Corona Survey; SCS) conducted 3–10 months later, between June and August 2020 (Scherpenzeel et al., 2020; Börsch-Supan, 2022h), *N* = 56 689. Participants were included if they also had cognitive test data from at least two waves including Wave 8 (*N* = 32 321), were above 50 years of age upon entering SHARE (*N* = 31 236), and had no missing pre–and peri-pandemic loneliness data (*N* = 31 148). Participants with an interval of more than 10 months between Wave 8 and the SCS (*N* = 1), who reported having been hospitalized or tested positive for COVID-19 (*N* = 155), and/or did not have a calibrated inverse probability weight for being part of the SCS subsample (*N* = 4) were excluded. Weights are provided as part of the SCS dataset (Scherpenzeel et al., 2020; Börsch-Supan, 2022h) and detailed information about the weights can be found in the SHARE Wave 8 and Corona Survey methodology documentation (De Luca et al., 2022; De Luca and Li Donni, 2022). The final sample consisted of participants who also fit into any of the three longitudinal loneliness groups of interest defined: (1) onset of loneliness from before to after the outbreak of COVID-19, (2) stable loneliness levels at both time points, and (3) no peri-pandemic loneliness (*N* = 30 245). Data from the same sample in SHARE waves 1–2 and 4–7 (Gruber et al., 2014; Bergmann et al., 2019; Börsch-Supan, 2022a,b,c,d,e,f), and a second SCS, conducted in June–August 2021 (Börsch-Supan, 2022i), was also used. For an overview of the SHARE time points, loneliness measures and groupings used in this study, see Figure 1.

SHARE data was supplemented with country-level pandemic-related data from the Oxford Coronavirus Government Response Tracker (OxCGRT; Hale et al., 2021, retrieved on 7th December 2021) and the COVID-19 Data Repository by the Center for Systems Science and Engineering (CSSE) at Johns Hopkins University (Dong et al., 2020, retrieved on 2nd Feburary 2022).

### 2.2. Measures

#### 2.2.1. Loneliness

Loneliness was measured using a single item asking participants to rate the general frequency with which they experienced feelings of loneliness on a three-point scale (often / some of the time / hardly ever or never), and defined as reporting feeling lonely at least some of the time. Based on this dichotomous loneliness measure, three groups were defined based on loneliness levels before (Wave 8) and during the pandemic (in the SCS): situational loneliness, persistent loneliness, and no loneliness. Situational loneliness (onset) was defined as no loneliness before the pandemic (in Wave 8), and loneliness during the pandemic (in the SCS). Note, due to the lack of a follow-up measure, we refer to this as situational rather than transient loneliness. Persistent loneliness (maintenance) was defined as identical loneliness ratings before and during the pandemic. Due to the relatively short time in between the measures, this is referred to as persistent rather than chronic loneliness. The third group did not report feeling lonely during the pandemic. Groups defined using the single-item measure were validated using scores on the Three-Item Loneliness Scale (Hughes et al., 2004), a short version of the Revised UCLA Loneliness Scale (Russell et al., 1980) that was used in SHARE waves 5–8. Both measures have been shown to be reliable and valid measures of loneliness and show good convergent validity (Mund et al., 2022).

In the SCS, participants who reported feeling lonely at least some of the time were asked to rate loneliness change since the outbreak of COVID-19 (“Has that been more so, less so or about the same as before the outbreak of Corona?”), and this data was used to compare self-rated retrospective loneliness increase to single-item longitudinal loneliness increase from Wave 8 to the SCS.

#### 2.2.2. Geographic region

European countries were grouped into regions according to the United Nations geoscheme for Europe (United Nations, 2021), and Israel was treated as a distinct region. Western European countries were Austria, Belgium, France, Germany, Luxembourg, Netherlands, and Switzerland. Northern European countries were Denmark, Estonia, Finland, and Sweden. Eastern European countries were Bulgaria, Czech Republic, Hungary, Latvia, Lithuania, Poland, Romania, and Slovakia. Southern Europe countries were Cyprus, Croatia, Greece, Italy, Malta, Slovenia, and Spain. For all participants, country of residence stayed the same from Wave 8 to the SCS.

#### 2.2.3. Demographic variables

Demographic variables included age in years, sex, migrant status, and nursing home residence. The 1997 International Standard Classification of Education (ISCED-97; United Nations Educational, Scientific and Cultural Organization [UNESCO], 2003) education level was trichotomized into less than upper secondary education, upper secondary including post-secondary non-tertiary education, and tertiary education. Area of residence was also trichotomized, comprising big city, large town or suburb, and small town or village. All demographic variables were measured at Wave 8, except for education level which was measures in each participant’s baseline wave, and age which was determined from the timepoint of the SCS, measured between March 3rd and July 31st 2020.

#### 2.2.4. Social network

Network size was defined as the number of persons that the participant listed in response to the interviewer asking who they have most often discussed important things during the past year (range 0–7), trichotomized into small network (0–1), medium network (2–3), and large network (4–7). Network satisfaction was measured by a single item asking participants how satisfied they were with their reported social network, on a scale from 0 to 10 (completely dissatisfied to completely satisfied). Low network satisfaction was defined as a rating below 8, capturing the bottom quartile of the population. Engagement in social activities was measured by four questions asking the participant how often they had done voluntary/charity work, attended an educational or training course, gone to a sport/social/other kind of club, or taken part in a political/community-related organization during the past year (almost daily, almost every week, almost every month, or less often). Not partaking in social activities was defined as not engaging in any of the activities with a frequency of at least “almost every week” during the past year. Further social network variables of interest were cohabiting with a partner (yes/no), widower status (yes/no), and having children (yes/no). All social network variables were measured or updated in Wave 8.

#### 2.2.5. Physical and cognitive health variables

Self-reported dichotomous health measures were two or more chronic diseases, limitations with at least one of seven Instrumental Activities of Daily Living (IADL; Lawton and Brody, 1969; Steel et al., 2002), engaging in vigorous activities such as sports, heavy housework, or a job that involves physical labor less than once a month, and having six or more alcoholic drinks at least three times per week. Body Mass Index (Quetelet, 1832) was trichotomized using the standard categories defined by the World Health Organization (World Health Organization [WHO], 1995): underweight (< 18.5), normal, and overweight (≥25). Depression was defined as a score above the cut off on the EURO-D depression scale (Prince et al., 1999; ≥4 points). A cognitive function sum score was computed using immediate and delayed word recall tests of verbal learning and memory, and a serial sevens subtraction test of working memory. Cognitive impairment was defined as a cognitive function sum score at least one standard deviation below the country mean at Wave 8, in combination with longitudinal decline (negative word recall slope across at least two time points, measured in Wave 8, and at least one previous wave). All other health variables were measured at Wave 8. Computation and validation of the cognitive impairment variable is detailed in Supplementary material.

#### 2.2.6. Pandemic variables

Pandemic-related country-level variables were the number of COVID-19 attributed deaths per million inhabitants, and number of months where extensive restrictions were in place in long term care facilities, and/or all older individuals were required to stay at home and not leave the home with minimal exceptions, and receive no external visitors (Phillips and Tatlow, 2021). Individual-level pandemic variables were reporting seldom having personal contact with others, defined as less than once a week, and reporting not having left one’s home since the start of the pandemic. Individual-level pandemic-related variables were measured at the SCS, and country-level variables were determined from the period of the SCS data collection, between March 3rd and July 31st 2020.

### 2.3. Statistical analyses

#### 2.3.1. Imputation of missing values and non-response adjustments

The proportion of missingness was 8.8% for the full dataset, with 4.16% for area of residence, 1.74% for BMI, and less than 1% for each of the other variables. Imputation of missing values was performed using predictive mean matching, prior to dichotomization or categorization of variables. Analyses were weighted using the cross-sectional calibrated inverse probability weights provided as part of the SCS dataset (Scherpenzeel et al., 2020; Börsch-Supan, 2022h). Individual weights reflect each responder’s probability of being part of the 2020 SCS sample, based on characteristics of the national target population, and weighted data results in more representative estimates. The weight calibration procedure in SHARE is described in detail by De Luca et al. (2022) and De Luca and Li Donni (2022).

#### 2.3.2. Validation of loneliness groupings

In order to partially validate the groupings that were defined by combining the single-item current loneliness scores from before and during the pandemic, mean scores on the Three-Item Loneliness Scale for the three groups were compared across the time points where this measure was also available (i.e., SHARE waves 5–8, not the SCSs), using a two-way mixed ANOVA for repeated measures. In addition, the longitudinal loneliness increase from Wave 8 to the SCS based on current reports was compared to the self-rated retrospective loneliness change from the SCS using a Chi-Square test of Independence.

#### 2.3.3. Predictors of situational and persistent loneliness

Three weighted hierarchical binary logistic regression analyses were performed. In Analysis 1, the outcome was situational over no loneliness (coded as 1 and 0, respectively). In Analysis 2, the outcome was persistent (1) over no loneliness (0), and in Analysis 3, the outcome was persistent (1) over situational loneliness (0).

Predictor candidates and control variables were identical across the analyses and were grouped in five blocks added one at a time: 1–geographic region, 2–demographic variables, 3–pre-pandemic social network, 4–pre-pandemic health, and 5–pandemic variables. Block 1 contained categorial Northern, Southern, and Eastern Europe, Israel, and Western Europe as reference. Block 2 comprised continuous age in years, sex with male as reference, categorical education level with upper secondary as reference, dichotomous migrant status and nursing home residence, and categorical residential area consisting of big city, rural area or small town, and large town or suburb as reference. Block 3 included dichotomous cohabitant partner, widow/-er status, having no children, engaging in no social activities, and having low network satisfaction. It also contained categorical network size, comprising small, large, and medium as reference. Block 4 contained dichotomous two or more chronic diseases, one or more IADL limitations, not engaging in any vigorous physical activities, alcohol consumption, cognitive impairment, and depression, as well as categorical BMI (underweight, overweight, and normal as reference). Block 5 included continuous deaths per million inhabitants, and number of older population isolation months, as well as dichotomous staying home, and seldom having personal contact with others, since the pandemic started. In order to account for practice effects (i.e., responses that might change as a function of exposure), control variables of no interest were categorical ordinary SHARE wave appearances (range 2–7, mode: 5, and including Wave 8), and continuous months between Wave 8 and SCS (range 3–10, M: 5.92, and SD: 1.45).

This procedure resulted in five regression models for each of the three analyses. For every block added, the aggregated model was compared to the previous model using the Rao-Scott Likelihood Ratio Chi-Squared Test. Model fit was assessed by the percentage of residuals falling within error bounds of ± 2 standard deviations (Gelman and Hill, 2006). Results are presented with robust standard errors, as returned by the “svyglm” function of the Survey package for R (Lumley, 2020), allowing for the identification of first order trends in the data. The Area Under the Receiver Operating Characteristic (AUC) was used to measure discriminative capacity.

#### 2.3.4. Software

Analyses were computed using RStudio version 1.4.1717, running on Mac OS X 12.3.1. Imputation of missing values was done using the Mice package (van Buuren and Groothuis-Oudshoorn, 2011), and regression analyses were done using the Survey package (Lumley, 2020).

## 3. Results

### 3.1. Descriptive statistics

**FIGURE 2:**
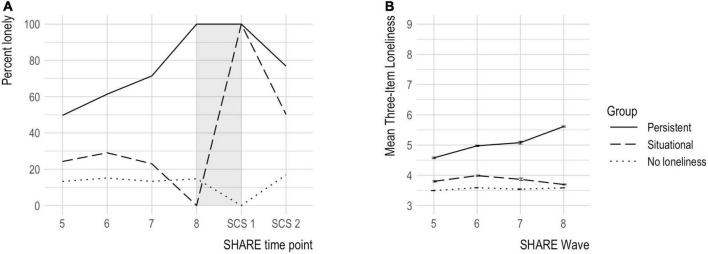
Loneliness in three groups over all available time points. **(A)** Percentage of groups reporting feeling lonely at ordinary SHARE wave 5 (*N* = 17 246), 6 (*N* = 21 508), 7 (*N* = 5 458), and 8 (*N* = 30 245), the first SHARE Corona Survey (SCS; SCS 1, *N* = 30 245), and the second SCS conducted 1 year later (SCS 2, *N* = 26 438). The gray band represents the period used to define the groups (Wave 8–SCS 1). **(B)** Mean Three-Item Loneliness Scale score (min = 3 and max = 9) and standard error for loneliness groups at ordinary SHARE wave 5 (*N* = 17 213), 6 (*N* = 21 487), 7 (*N* = 5 433), and 8 (*N* = 30 176).

The final sample consisted of 30 245 older adults, 56.5% female, between 52 and 104 years of age (M: 70.33 and SD: 8.64). Sample characteristics for SHARE variables are presented in Table 1. Across countries, the average older population isolation period lasted for 2.17 months (SD: 1.16, median: 3.80, and range: 0–5.03), and the mean number of COVID-19 deaths per million inhabitants was 184.65 (SD: 233.74, median: 35.53, and range: 5.32–846). Figure 2A shows longitudinal loneliness incidence based on the single-item cut off, defined between Wave 8 and SCS 1, for all waves.

**TABLE 1:** Sample characteristics for categorical and dichotomous SHARE variables, by loneliness group.

Variable	No loneliness		Situational		Persistent	
	Total	%	Total	%	Total	%
Western Europe	6 914	30.9	1 007	27.3	1 020	24.3
Northern Europe	4 390	19.6	592	16.1	616	14.6
Southern Europe	5 292	23.7	1 094	29.7	1 421	33.8
Eastern Europe	5 352	23.9	932	25.3	1 080	25.7
Israel	409	1.8	52	1.5	69	1.6
Sex (female)	11 771	52.7	2 420	65.7	2 905	69.1
Lower education	5 905	26.4	1 223	33.2	1 653	39.3
Upper secondary education	10 531	47.1	1 614	43.8	1 764	41.9
Higher education	5 921	26.5	845	22.9	789	18.8
Migrant	1 812	8.1	316	8.6	371	8.8
Nursing home	90	0.4	30	0.8	46	1.1
AoR 1 (big city)	3 717	16.6	662	18.0	787	18.7
AoR 2 (large town/suburb)	5 497	24.6	942	25.6	1 104	26.2
AoR 3 (rural/small town)	13 143	58.8	2 078	56.4	2 315	55.0
No cohabitant partner	5 159	23.1	1 470	39.9	2 668	63.4
Widowed	2 805	12.5	838	22.8	1 640	39.0
No children	1 617	7.2	327	8.9	546	13.0
Medium network	10 278	46.0	1 730	47.0	1 981	47.1
Small network	5 500	24.6	950	25.8	1 313	31.2
Large network	6 579	29.4	1 002	27.2	912	21.7
No social activities	15 922	71.2	2 818	76.5	3 415	81.2
Low network satisfaction	1 595	7.1	336	9.1	741	17.6
2+ chronic diseases	11 149	49.9	2 119	57.6	2 673	63.6
IADL limitations	2 819	12.6	726	19.7	1 199	28.5
BMI (normal)	7 120	31.8	1 187	32.2	1 358	32.2
BMI (underweight)	214	1.0	37	1.0	63	1.5
BMI (overweight)	15 023	67.2	2 458	66.8	2 785	66.2
No physical activities	8 524	38.1	1 671	45.4	2 142	50.9
Alcohol	904	4.0	113	3.1	143	3.4
Cognitive impairment	1 910	8.5	372	10.1	664	15.8
EURO-D depression	4 177	18.7	1 060	28.8	2 135	50.8
Stayed home since outbreak	2 772	12.4	713	19.4	953	22.2
Seldom personal contact	7 691	34.4	1 368	37.2	1 517	36.1

AoR, area of residence.

### 3.2. Loneliness grouping validation and comparison to retrospective self-rated change

There was a statistically significant interaction between loneliness group and wave number for the Three-Item Loneliness Scale score, *F*_(6, 11 700)_ = 77.59, *p* = < 0.0001. The main effect of loneliness group was significant at all-time points [*p* = < 0.0001 (Wave 5), *p* = < 0.0001 (Wave 6), *p* = < 0.0001 (Wave 7), and *p* = < 0.0001 (Wave 8)], and pairwise comparisons showed that the mean Three-Item Loneliness Scale score was significantly different between all groups at all-time points (all *p* = < 0.0001; for details, see Supplementary Table 5). Figure 2B shows longitudinal mean scores on the Three-Item Loneliness Scale for all available time points (SHARE waves 5–8) for the three loneliness groups.

Interestingly, the single-item self-rated retrospective loneliness increase at the SCS did not correspond to longitudinal single-item loneliness increase from Wave 8 to the SCS, χ^2^ (1, *N* = 7 888) = 34.64, *p* < 0.001, with loneliness increase being reported by 42.5% of the situational loneliness group, characterized by an increase in longitudinal loneliness, and 36.0% of the persistent loneliness group characterized by stable longitudinal loneliness levels.

### 3.3. Predictors of situational loneliness

The first hierarchical regression analysis identified predictors of situational loneliness, i.e., onset, compared to no loneliness. Essential results of Analysis 1 are presented in Table 2, and full results including data for variables that were non-significant in all models can be found in Supplementary Table 6. Consistent predictors across all blocks were female sex, no cohabitant partner, two or more chronic diseases, and depression. Staying home and seldom having personal contact with others since the outbreak of COVID-19 were both associated with higher, while frequent alcohol use was associated with lower odds of situational loneliness. Chronological age was significant when adding the second block (Model 2) but rendered insignificant after social network variables were added. Eastern and Southern Europe predicted situational loneliness until pandemic variables were added, suggesting that these relationships may have been mediated by pandemic-related factors. Living in Northern Europe was a protective factor in the final model. For the full model, 96% of binned residuals were within error bounds, and discriminative ability was poor (AUC = 0.65).

**TABLE 2:** Analysis 1, outcome: Situational loneliness (1) – No loneliness (0).

Variable	Model 1					Model 2					Model 3					Model 4					Model 5				
	Coef.	Se	OR	95% CI	Sig.	Coef.	Se	OR	95% CI	Sig.	Coef.	Se	OR	95% CI	Sig.	Coef.	Se	OR	95% CI	Sig.	Coef.	Se	OR	95% CI	Sig.
Intercept	**−2.48**	**0.29**	**0.08**	**0.05–0.15**	***	**−3.45**	**0.47**	**0.03**	**0.01–0.08**	***	**−3.27**	**0.51**	**0.04**	**0.01–0.10**	***	**−2.95**	**0.58**	**0.05**	**0.02–0.16**	***	**−2.85**	**0.54**	**0.06**	**0.02–0.17**	***
**BLOCK 1: Geographic region**																									
Northern Europe	−0.15	0.11	0.86	0.70–1.06		−0.21	0.11	0.81	0.66–1.00		−0.20	0.11	0.82	0.67–1.01		−0.18	0.11	0.84	0.68–1.03		**−0.27**	**0.14**	**0.76**	**0.58**–**1.00**	*
Southern Europe	**0.46**	**0.15**	**1.58**	**1.18**–**2.11**	**	**0.45**	**0.14**	**1.58**	**1.21**–**2.05**	***	**0.46**	**0.14**	**1.58**	**1.20**–**2.07**	***	**0.50**	**0.14**	**1.65**	**1.26**–**2.15**	***	0.21	0.21	1.23	0.82–1.84	
Eastern Europe	**0.38**	**0.12**	**1.47**	**1.17**–**1.85**	**	**0.32**	**0.12**	**1.38**	**1.09**–**1.74**	**	**0.28**	**0.13**	**1.32**	**1.03**–**1.69**	*	**0.29**	**0.13**	**1.33**	**1.04**–**1.71**	*	0.23	0.13	1.26	0.98–1.63	
**BLOCK 2: Demographic variables**																									
Age						**0.01**	**0.01**	**1.01**	**1.00**–**1.02**	*	0.01	0.01	1.01	0.99–1.02		0.00	0.01	1.00	0.99–1.01		0.00	0.01	1.00	0.98–1.01	
Sex (female)						**0.64**	**0.10**	**1.90**	**1.57**–**2.31**	***	**0.54**	**0.10**	**1.72**	**1.41**–**2.10**	***	**0.47**	**0.11**	**1.60**	**1.30**–**1.98**	***	**0.46**	**0.10**	**1.58**	**1.29**–**1.93**	***
**BLOCK 3: Objective and subjective social network variables**																									
No cohabitant partner											**0.45**	**0.13**	**1.57**	**1.22–2.00**	***	**0.44**	**0.13**	**1.55**	**1.20–1.98**	***	**0.47**	**0.13**	**1.60**	**1.25–2.05**	***
**BLOCK 4: Physical and cognitive health variables**																									
2+ chronic diseases																**0.24**	**0.09**	**1.27**	**1.06–1.52**	*	**0.25**	**0.09**	**1.28**	**1.07–1.54**	**
Alcohol																**−0.54**	**0.17**	**0.58**	**0.41–0.82**	**	**−0.60**	**0.18**	**0.55**	**0.39–0.78**	***
Depression																**0.29**	**0.09**	**1.34**	**1.12–1.60**	**	**0.28**	**0.09**	**1.33**	**1.10–1.59**	**
**BLOCK 5: Pandemic variables**																									
Stayed home																					**0.42**	**0.21**	**1.53**	**1.01–2.31**	*
Seldom personal contact																					**0.30**	**0.10**	**1.35**	**1.11–1.65**	**

Variables not shown (non-significant in all models): BLOCK 1: Israel, BLOCK 2: education level, migrant status, nursing home, residential area, BLOCK 3: widower status, no children, social network size, no social activities, low network satisfaction, BLOCK 4: IADL limitations, no vigorous physical activities, cognitive impairment, Body Mass Index categories, BLOCK 5: deaths per million inhabitants, isolation months. Full table with data for all variables can be found in Supplementary Table 6. Significance levels: *p < 0.05, **p < 0.01, ***p < 0.001. Bold characters indicate statistical significance in the model.

### 3.4. Predictors of persistent loneliness

The second analysis identified predictors of persistent loneliness, i.e., maintenance, compared to no loneliness. Essential results of Analysis 2 are presented in Table 3, and full results including data for variables that were non-significant in all models can be found in Supplementary Table 7. Consistent predictors of loneliness maintenance across all blocks were female sex, low education, no cohabitant partner, low network satisfaction, one or more IADL limitations, depression, and widowhood. Living in a country with a longer isolation period for older individuals, and a lower number of deaths attributed to COVID-19 per million inhabitants, also predicted persistent loneliness. Residing in a nursing home or small town/rural area, both protective, became significant after the inclusion of health and pandemic variables, respectively. Chronic diseases predicted persistent loneliness after the inclusion of pandemic variables, and chronological age was rendered insignificant when health variables were added. Living in Eastern Europe was a consistent predictor of persistent loneliness, while Southern Europe was significant until pandemic variables were added. Living in Northern Europe or Israel were protective factors in the final model. For the full model, 93% of binned residuals fell within error bounds, and discriminative ability was excellent (AUC = 0.81).

**TABLE 3:** Analysis 2, outcome: Persistent loneliness (1) – No loneliness (0).

Variable	Model 1					Model 2					Model 3					Model 4					Model 5				
	Coef.	Se	OR	95% CI	Sig.	Coef.	Se	OR	95% CI	Sig.	Coef.	Se	OR	95% CI	Sig.	Coef.	Se	OR	95% CI	Sig.	Coef.	Se	OR	95% CI	Sig.
Intercept	**−2.28**	**0.20**	**0.10**	**0.07–0.15**	***	**−3.27**	**0.38**	**0.04**	**0.02–0.08**	***	**−4.07**	**0.41**	**0.02**	**0.01–0.04**	***	**−3.46**	**0.45**	**0.03**	**0.01–0.08**	***	**−4.33**	**0.48**	**0.01**	**0.01–0.03**	***
**BLOCK 1: Geographic region**																									
Northern	−0.13	0.11	0.87	0.70–1.09		−0.17	0.12	0.84	0.67–1.06		−0.09	0.12	0.91	0.72–1.16		0.01	0.12	1.01	0.80–1.27		**−0.31**	**0.13**	**0.73**	**0.56–0.95**	*
Southern	**0.39**	**0.10**	**1.47**	**1.20–1.80**	***	**0.28**	**0.10**	**1.32**	**1.08–1.62**	**	**0.35**	**0.12**	**1.43**	**1.13–1.79**	**	**0.43**	**0.12**	**1.53**	**1.20–1.96**	***	−0.23	0.20	0.79	0.53–1.18	
Eastern	**0.46**	**0.11**	**1.58**	**1.26–1.97**	***	**0.34**	**0.11**	**1.40**	**1.13–1.75**	**	**0.30**	**0.13**	**1.35**	**1.05–1.74**	*	**0.29**	**0.14**	**1.34**	**1.03–1.75**	*	**0.28**	**0.14**	**1.33**	**1.01–1.75**	*
Israel	0.30	0.40	1.35	0.62–2.93		0.02	0.38	1.02	0.48–2.16		0.07	0.35	1.07	0.53–2.15		0.05	0.42	1.05	0.46–2.38		**−0.95**	**0.47**	**0.39**	**0.15–0.97**	*
**BLOCK 2: Demographic variables**																									
Age						**0.03**	**0.00**	**1.03**	**1.02–1.04**	***	**0.01**	**0.00**	**1.01**	**1.00–1.02**	*	0.00	0.01	1.00	0.99–1.01		0.00	0.01	1.00	0.99–1.01	
Sex (female)						**0.76**	**0.08**	**2.13**	**1.82–2.49**	***	**0.46**	**0.09**	**1.59**	**1.33–1.90**	***	**0.26**	**0.09**	**1.29**	**1.08–1.56**	**	**0.24**	**0.09**	**1.27**	**1.06–1.53**	*
Lower edu.						**0.41**	**0.09**	**1.50**	**1.26–1.79**	***	**0.42**	**0.10**	**1.52**	**1.25–1.84**	***	**0.31**	**0.10**	**1.37**	**1.12–1.67**	**	**0.34**	**0.11**	**1.41**	**1.14–1.73**	**
Nursing h.						0.07	0.33	1.07	0.56–2.06		−0.43	0.33	0.65	0.34–1.25		**−0.70**	**0.32**	**0.50**	**0.27–0.93**	*	**−0.75**	**0.32**	**0.47**	**0.25–0.89**	*
AoR 3 (rural/small)						**−0.31**	**0.10**	**0.74**	**0.60–0.91**	**	−0.19	0.11	0.82	0.66–1.03		−0.23	0.12	0.80	0.63–1.00		**−0.25**	**0.12**	**0.78**	**0.62–0.98**	*
**BLOCK 3: Objective and subjective social network variables**																									
No cohab. partner											**1.47**	**0.11**	**4.33**	**3.48–5.39**	***	**1.40**	**0.11**	**4.07**	**3.26–5.09**	***	**1.44**	**0.11**	**4.21**	**3.36–5.27**	***
Widowed											**0.36**	**0.11**	**1.44**	**1.16–1.78**	***	**0.39**	**0.11**	**1.48**	**1.18–1.85**	***	**0.38**	**0.12**	**1.47**	**1.17–1.84**	***
Low net. satisfaction											**0.95**	**0.12**	**2.57**	**2.02–3.28**	***	**0.81**	**0.13**	**2.25**	**1.76–2.88**	***	**0.80**	**0.13**	**2.22**	**1.73–2.84**	***
**BLOCK 4: Physical and cognitive health variables**																									
2+ chronic																0.17	0.09	1.19	0.99–1.43		**0.19**	**0.09**	**1.21**	**1.01–1.46**	*
IADL lim.																**0.32**	**0.11**	**1.38**	**1.11–1.72**	**	**0.30**	**0.11**	**1.35**	**1.09–1.69**	**
Depression																**1.26**	**0.09**	**3.54**	**2.98–4.20**	***	**1.27**	**0.09**	**3.55**	**2.99–4.22**	***
**BLOCK 5: Pandemic variables**																									
Deaths																					**−0.00**	**0.00**	**1.00**	**1.00–1.00**	**
Isolation months																					**0.69**	**0.14**	**2.00**	**1.51–2.66**	***
Seldom personal contact																					**0.17**	**0.09**	**1.19**	**1.00–1.41**	*

AoR, area of residence; edu., education; nursing h., nursing home; cohab., cohabitant; net., network; lim., limitations. Variables not shown (non-significant in all models): BLOCK 2: higher education (tertiary), migrant status, residential area 2 (large town/suburb), BLOCK 3: no children, social network size, no social activities, BLOCK 4: no vigorous physical activities, frequent alcohol consumption, cognitive impairment, Body Mass Index categories, BLOCK 5: staying home since the pandemic outbreak. Full table with data for all variables can be found in Supplementary Table 7. Significance levels: *p < 0.05, **p < 0.01, ***p < 0.001. Bold characters indicate statistical significance in the model.

### 3.5. Predictors of persistent over situational loneliness

The third analysis identified predictors of persistent loneliness compared to situational loneliness, and the essential results of Analysis 3 are presented in Table 4. Full results including data for variables that were non-significant in all models can be found in Supplementary Table 8. Consistent predictors of persistent over situational loneliness across all blocks were no cohabitant partner, low network satisfaction, one or more IADL limitations, frequent alcohol use, depression, and living in a country with a longer isolation period for older individuals. Chronological age and low education were significant when adding the second block (Model 2), but rendered insignificant when social network variables were added. For the full model, 97% of binned residuals fell within error bounds, and discriminative ability was acceptable (AUC = 0.70).

**TABLE 4:** Analysis 3, outcome: Persistent loneliness (1) – Situational loneliness (0).

Variable	Model 1					Model 2					Model 3					Model 4					Model 5				
	Coef.	Se	OR	95% CI	Sig.	Coef.	Se	OR	95% CI	Sig.	Coef.	Se	OR	95% CI	Sig.	Coef.	Se	OR	95% CI	Sig.	Coef.	Se	OR	95% CI	Sig.
Intercept	**0.21**	**0.31**	**1.23**	**0.67–2.27**	*	−1.02	0.51	0.36	0.13–0.99		−0.92	0.56	0.40	0.13–1.19		−0.77	0.58	0.46	0.15–1.45		−1.10	0.62	0.33	0.10–1.11	
**BLOCK 2: Demographic variables**																									
Age						**0.02**	**0.01**	**1.02**	**1.01–1.03**	**	0.00	0.01	1.00	0.99–1.02		0.00	0.01	1.00	0.98–1.01		0.00	0.01	1.00	0.98–1.01	
Lower education						**0.26**	**0.13**	**1.30**	**1.02–1.66**	*	0.24	0.12	1.27	1.00–1.61		0.11	0.12	1.12	0.88–1.42		0.14	0.12	1.15	0.90–1.45	
**BLOCK 3: Objective and subjective social network variables**																									
No cohabitant partner											**1.05**	**0.15**	**2.86**	**2.13–3.84**	***	**1.07**	**0.15**	**2.91**	**2.16–3.92**	***	**1.05**	**0.15**	**2.87**	**2.13–3.87**	***
Low network satisfaction											**0.86**	**0.15**	**2.37**	**1.78–3.16**	***	**0.72**	**0.15**	**2.06**	**1.54–2.75**	***	**0.71**	**0.15**	**2.04**	**1.53–2.74**	***
**BLOCK 4: Physical & cognitive health variables**																									
IADL limitations																**0.31**	**0.12**	**1.36**	**1.08–1.73**	*	**0.34**	**0.12**	**1.40**	**1.10–1.78**	**
Alcohol																**0.68**	**0.31**	**1.97**	**1.07–3.63**	*	**0.67**	**0.30**	**1.96**	**1.08–3.56**	*
Depression																**1.04**	**0.10**	**2.82**	**2.30–3.47**	***	**1.04**	**0.10**	**2.83**	**2.30–3.47**	***
**BLOCK 5: Pandemic variables**																									
Isolation months																					**0.21**	**0.09**	**1.24**	**1.03–1.48**	*

Variables not shown (non-significant in all models): BLOCK 1: all geographic regions, BLOCK 2: female sex, higher education (tertiary), migrant status, nursing home, residential area, BLOCK 3: widower status, no children, social network size, no social activities, BLOCK 4: two or more chronic diseases, no vigorous physical activities, cognitive impairment, Body Mass Index categories, BLOCK 5: deaths per million inhabitants, staying home since the pandemic outbreak, seldom having personal contact with others. Full table with data for all variables can be found in Supplementary Table 8. Significance levels: *p < 0.05, **p < 0.01, ***p < 0.001. Bold characters indicate statistical significance in the model.

**FIGURE 3:**
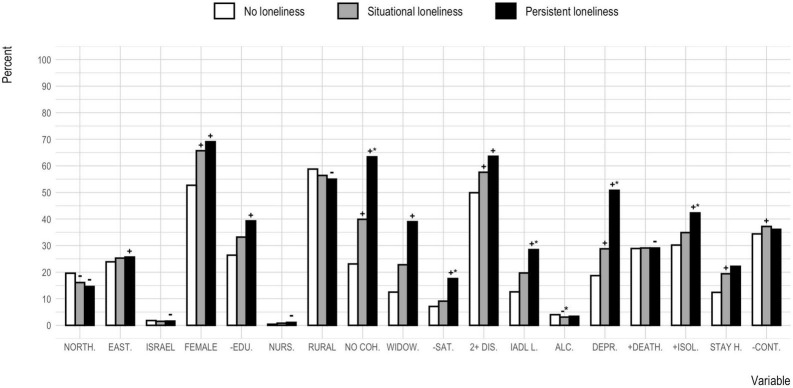
Frequency, significance, and direction of association for all significant variables in the full model from analyses 1, 2, and 3, per loneliness group. The continuous country-level variables deaths per million and number of older population isolation months have been dichotomized to reflect frequency of above average observations. NORTH., Northern Europe; EAST., Eastern Europe; FEMALE, female sex; -EDU, less than upper secondary education; NURS., residing in nursing home; RURAL, residing in rural area or small town; NO COH., no cohabitant partner; WIDOW, widowed; -SAT., low network satisfaction; 2 + DIS., two or more chronic diseases; IADL L., one or more IADL limitations; ALC., having six or more alcoholic drinks at least three times per week; DEPR., above cut-off on the EURO-D Depression Scale; + DEATH., above average COVID-19 attributed deaths per million inhabitants; + ISOL., above average number of older population isolation months; STAY H., stayed home since the beginning of the pandemic; -CONT., seldom personal contact. ^+^Significantly higher odds for situational or persistent loneliness, as compared to the no loneliness group (in Analysis 1 or 2). ^–^Significantly lower odds for situational or persistent loneliness, as compared to the no loneliness group (in Analysis 1 or 2). *Significantly higher or lower (±) odds for persistent/situational loneliness, as compared to situational/persistent loneliness (in Analysis 3).

Results of all model comparisons are presented in Table 5. For all analyses, the addition of each covariate block resulted in a significant model improvement, except for the addition of demographic variables to the intercept and control variables model in Analysis 3. Frequency, significance, and direction of association for all significant variables in the full models from Analysis 1, 2, and 3 are shown in Figure 3.

**TABLE 5:** Results of Rao-Scott Likelihood Ratio Chi-Squared Tests for stepwise comparisons of the models when adding one additional block of covariates in analyses 1 (situational over no loneliness), 2 (persistent over no loneliness), and 3 (persistent over situational loneliness).

Comparison	Analysis 1			Analysis 2			Analysis 3		
	*df*	χ^2^	Sig.	*df*	χ^2^	Sig.	*df*	χ^2^	Sig.
Intercept + ctrl–Model 1	4	127.88	**	4	134.64	***	4	5.05	
Model 1–Model 2	8	424.22	***	8	1108.65	***	8	127.28	**
Model 2–Model 3	7	205.61	***	7	2513.35	***	7	723.48	***
Model 3–Model 4	8	136.56	*	8	1332.56	***	8	516.84	***
Model 4–Model 5	5	134.84	*	5	162.96	***	4	39.23	*

df, degrees of freedom. Significance levels: *p < 0.05, **p < 0.01, ***p < 0.001.

## 4. Discussion

Predictors of loneliness onset and maintenance among older adults in the COVID-19 pandemic were investigated among frequently studied candidate variables. Here, those individuals who reported feeling frequently lonely during the pandemic were further divided into groups of situational and persistent loneliness according to their reports of loneliness obtained before the pandemic. As hypothesized, hierarchical logistic regression analyses identified unique, shared, and pandemic-specific predictors of loneliness during the pandemic for those different groups.

This is in line with research showing that cross-sectional measures of loneliness capture both a remarkably stable trait and a more dynamic state component (Mund et al., 2020a,b). Indeed, the model for situational analysis showed worse discriminative ability than that for persistent loneliness, possibly reflecting the difficulty of predicting a state as compared to a trait. Comparing the persons with situational and persistent loneliness on other measures of loneliness, we found that a three-item loneliness measure obtained before the pandemic confirmed differences in loneliness as far back as 7 years prior to the pandemic. However, retrospective reports of subjective increases in loneliness at the time of the pandemic did not relate to longitudinal reports at each wave. Based on these and previous findings, and in line with our hypothesis, we argue that the group reporting cross-sectional loneliness is heterogeneous, containing both transient and chronic loneliness which should be treated as different constructs, at least until their similarities and differences have been fully established.

In contrast to prior work by Yang (2018), three unique predictors for persistent, and one for situational, loneliness were found. Among these was the country-level length of recommended or enforced isolation for older adults, uniquely predicting persistent loneliness. This variable has, to our knowledge, not been studied in relation to loneliness before. Extending previous findings (Atzendorf and Gruber, 2021; Buecker and Horstmann, 2021), this result indicates that pre-and peri-pandemic loneliness predictors were not identical when considering situational and persistent loneliness separately, and that restrictions and social policy had an impact on maintenance of loneliness in the older adult population during this time. Low network satisfaction and functional limitations were also unique predictors of persistent loneliness. While self-rated quality of social relationships has consistently been identified as a risk factor for loneliness in older adults, evidence regarding functional limitations has been ambiguous (Dahlberg et al., 2022). Functional limitations may have been more closely related to loneliness maintenance during the COVID-19 pandemic, or, alternatively, the disparity of previous findings may be due to the grouping together of persistent and situational loneliness.

Out of the five shared predictors of situational and persistent loneliness, two were also associated with significantly higher odds of loneliness maintenance rather than onset. These were depression and no cohabitant partner, each more than doubling the odds of experiencing persistent over both situational and no loneliness, with the largest effect sizes (small to medium) of any predictors in the study. Depression has consistently been associated with loneliness in longitudinal studies of older adults (Dahlberg et al., 2022), and the mutually aggravating relationship between loneliness and depression over time described by Luanaigh and Lawlor (2008) could possibly account for the stronger association to persistent loneliness found in this study. Marital status and cohabitation have also been consistent predictors of loneliness before and during the pandemic (Bu et al., 2020; Groarke et al., 2020; Hansen et al., 2021; O’Shea et al., 2021; van Tilburg et al., 2021; Dahlberg et al., 2022), although a greater influence of quality over quantity of social relationships has been argued (Pinquart and Sörensen, 2003). In this study, however, quality of relationships (network satisfaction) was only a significant predictor alongside quantity (cohabitant partner, but not network size) in the case of persistent loneliness. While lending further weight to partnership as a protective factor against loneliness in general, our findings point to the absence of a cohabiting partner as a particularly strong predictor of long-term loneliness. This is consistent with theoretical models of chronic loneliness as a self-sustaining, vicious circle of social withdrawal (Qualter et al., 2015), minimizing opportunities for meeting and engaging with potential partners.

Both situational and persistent loneliness were predicted by geographical region, physical health, and sex. The results for geographical regions were largely similar to findings from a pre-pandemic meta-analysis by Surkalim et al. (2022), with residents of Northern Europe being less likely than those of Western Europe to report feeling lonely. Since the evidence for chronic illness as a risk factor for loneliness was not found to be consistent in the review by Dahlberg et al. (2022), the relationship we found may be unique to the pandemic context, e.g., through belonging to a COVID-19 risk group. The sex difference found in this study is consistent with previous research using a direct single-item measure of loneliness (von Soest et al., 2018), with females being significantly more likely than males to report feeling lonely when asking the participant directly about how lonely they feel. However, females were also found to experience a significantly larger increase in loneliness from before to during the COVID-19 pandemic in a study by Entringer and Gosling (2022), using an indirect multi-item loneliness measure, not excluding the possibility of female sex being a pandemic-specific risk factor.

Inconsistently, two predictors of loneliness onset, and seven predictors of loneliness maintenance, were significant when compared to the non-loneliness group, but non-significant in the third comparison analysis. Further research is required in order to establish whether, or through which mechanisms, factors such as pandemic behavior, education, residential area, and widowhood act upon transient and chronic loneliness. In addition, the results showed that frequent alcohol consumption (having six or more alcoholic drinks at least three times per week during the past 3 months) was more common among both the persistent and no loneliness groups as compared to the situational loneliness group. This is a counter-intuitive finding that may be further studied in samples with a higher frequency of alcohol abuse, or including a more refined measure that distinguishes types of alcohol, multi-item scales, and structured interview instruments.

### 4.1. Strengths and limitations

To our knowledge, it is the first study to separately investigate predictors of situational and persistent loneliness during the COVID-19 pandemic from longitudinal data. We used a large sample from a longitudinal cross-country survey and were able to control for a large number of previously studied variables. Combining ordinary SHARE waves and the peri-pandemic SCS, we were able to rely on longitudinal self-reports rather than retrospective self-reported change which may introduce bias (Blome and Augustin, 2015; Hipp et al., 2020), e.g., if self-assessments are collectively impacted by changes in societal norms (Jaspers et al., 2009), and which did not correspond to longitudinal change in this sample. However, the single-item loneliness measure used in this study, while being adequate and comparable to more complex multi-item measures (Mund et al., 2022), does not allow for the distinction of social and emotional loneliness. Originally described by Weiss (1973), emotional loneliness refers to the lack of a close, intimate attachment to another person, while social loneliness denotes a perceived lack of social networks that provide a wider sense of belonging and community (Russell et al., 1984; de Jong Gierveld et al., 2006). These two facets of loneliness have different correlates (Dahlberg and McKee, 2014; Lee et al., 2022), and a need for further research distinguishing between predictors of emotional and social loneliness, respectively, has been pointed out (Dahlberg et al., 2022). In addition, these facets of loneliness may have been differently impacted by the pandemic, and a larger pandemic-related increase in emotional rather than social loneliness has been found by van Tilburg (2022). Future research should investigate facets of loneliness within the context of situational and persistent loneliness.

In-depth analysis of higher-order relationships, interactions, and mediation among predictor candidates was also outside the scope of this study. Given the investigation of loneliness onset and maintenance during a pandemic, our results may be either general or specific to these circumstances, and generalization of the findings requires corroboration outside of the pandemic context. Further, onset of loneliness in the COVID-19 pandemic likely represents the start of persistent feelings for some, and a passing state for others. The specific identification of predictors for onset of persistent loneliness would have required the use of more time points. This is, however, an important avenue for future research, as the development of chronic loneliness, leading to worse health outcomes than transient loneliness, is an important target for intervention.

### 4.2. Social policy recommendations

In our study, persistent loneliness in older adults was predicted by length of isolation during the first months of the COVID-19 pandemic. Under social contact restrictions, persistently lonely older adults may be seen as a particularly vulnerable group, and may also lack the internal and external resources to “bounce back” and attain social reconnection after a period of mandated social isolation. Therefore, future lockdowns and social contact restrictions should be followed up with efforts put in place in order to minimize their long-term negative consequences for this particular group. While severe circumstances such as the COVID-19 pandemic may increase the risk of loneliness maintenance among persistently lonely individuals, it remains imperative to target chronic loneliness during times when no restrictions are in place. Since the United Kingdom launched a national strategy to combat loneliness and became the first European country to appoint a Minister for Loneliness in 2018 (United Kingdom Department for Digital Culture, Media, and Sport, 2018), more light has been shed on loneliness as a major public health concern, and more national and European efforts have been directed toward combating loneliness in the aftermath of the COVID-19 pandemic [e.g., the 2021 Policy Brief titled “Addressing loneliness and social isolation among older people in Europe” (European Centre for Social Welfare Policy and Research, 2021), the 2022 German Strategy Against Loneliness (Federal Ministry for Family Affairs, Senior Citizens, Women and Youth, 2023), and the special focus on loneliness announced by the Swedish Minister for Social Affairs and Public Health as part of the Swedish 6 month presidency of the Council of the European Union in early 2023 (Swedish Presidency of the Council of the European Union, 2023)]. National strategies may seek to address loneliness risk factors on a country or municipality level, e.g., by instituting policies recommending screening for loneliness and specialized treatment of late-life depression in public health settings, shared housing developments for older citizens, and promoting research on, and dissemination of, effective treatment protocols specifically targeting the thoughts and behaviors that work to maintain chronic loneliness on an individual level.

## 5. Conclusion

Loneliness and its negative consequences for mental and physical wellbeing is an important facet of the impact of the COVID-19 pandemic on older adults. Our study shows that a distinction of situational and persistent loneliness is warranted. The sensitivity of persistent loneliness to length of isolation should be taken into account when employing social policies affecting older adults. Interventions aimed at preventing or addressing loneliness may target persons with symptoms of depression, functional limitations, chronic health issues, and no cohabitant partner. Future research should aim to develop a standard classification of transient and chronic loneliness, and to further the understanding of their unique or shared risk factors and outcomes. Where the temporal relationship is unclear, efforts should be made to understand the mechanisms by which persistent loneliness and its predictors are associated, e.g., whether these factors maintain chronic loneliness independently, or whether they mediate the relationship between prior and future loneliness. Apart from identifying risk groups, the characterization of such mechanisms may direct intervention targets, e.g., environmental circumstances, or individual patterns of thought and behavior.

## Data availability statement

Publicly available datasets were analyzed in this study. This data can be found here: https://share-eric.eu/data/data-documentation/share-data-releases.

## Ethics statement

The studies involving human participants were reviewed and approved by the Ethics Committee of the University of Mannheim (SHARE waves 1 to 3) and the Ethics Council of the Max Planck Society (SHARE waves 4 onward). For more details please see: https://share-eric.eu/fileadmin/user_upload/Ethics_Documentation/SHARE_ethics_approvals.pdf. Written informed consent for participation was not required for this study in accordance with the national legislation and the institutional requirements.

## Author contributions

VPL and AR designed the research. VPL and MJ analyzed the data. VPL wrote the manuscript. AR and MJ contributed to the writing. All authors contributed to the article and approved the submitted version.
