# Supplementary material for: Predictors of loneliness onset and maintenance in European older adults during the COVID-19 pandemic
Source: Front Psychol. 2023 Jun 2;14:1172552. doi: 10.3389/fpsyg.2023.1172552 (PMC10272806; doi:10.3389/fpsyg.2023.1172552)
Supplement: Supplementary file 1 [file Data_Sheet_1.pdf]

Supplementary Material

Predictors of Loneliness Onset and Maintenance in European Older Adults During the Covid-19 Pandemic

Vania Panes Lundmark*, Maria Josefsson, Anna Rieckmann*

*** Correspondence:**
Vania Panes Lundmark: vania.panes-lundmark@umu.se
Anna Rieckmann: anna.rieckmann@unibw.de

1 Computation of a cognitive function sum score and validation of a country-dependent cut off score for cognitive impairment

1.1 Measures of cognitive function & classification of cognitive impairment

Individuals with age-related cognitive impairment are expected to perform below average on tests of memory and executive function, and to show a decline in cognitive performance over time (Ahmadi-Abhari et al., 2017). In this study, a cognitive sum score cut-off at one time point was combined with individual memory performance development across several time points in order to classify cognitive impairment.

1.1.1 Sample

The computation of a cognitive function sum score, and the establishment of a cut-off for this score, was done using the full sample of participants from Wave 8, above the age of 50 upon study entry, and with no missing data for the relevant tests (N = 39 945, 22 318 women, 55.9 %, age range: 50 – 103 years, m: 70.07, SD: 8.79), irrespective of participation in the Covid-19 Survey. Out of these 39 945 individuals, definition a dichotomous cognitive impairment variable was possible for 39 209 individuals (22 008 women, 56.1 %, age range: 52 – 103 years, m: 70.13, SD: 8.78), who also had word recall data from at least one additional SHARE Wave.

1.1.2 Cognitive function sum score

The cognitive function sum score comprises three tests from the SHARE cognitive function module: the immediate and delayed word recall tests of verbal learning and memory, and the serial sevens subtraction test of working memory. The tests are all part of a cognition summary score previously evaluated within the Health and Retirement Study (Langa, 2020), that has been shown to adequately identify and distinguish between levels of cognitive impairment (Crimmins et al., 2011).

In the SHARE word recall test, participants were read a list of 10 nouns. For waves 1 and 2, the word list was the same across participants, while in waves 4 – 8, participants were randomly assigned one of four different lists. After being presented with the list, participants were asked to recall as many of the words as possible, within a time frame of one minute (immediate recall). After engaging in verbal fluency and numeracy tests during a delay time of 5 – 10 minutes, participants were again allowed up to one minute to recall as many of the words as possible (delayed recall). One point was

awarded for every word correctly recalled (max 10 points for immediate and delayed recall, respectively).

In the serial sevens test, participants were asked to count backwards from one hundred by subtracting seven at a time, and stopping after the fifth answer. One point per answer was awarded if the number given was the correct difference of the previous answer and seven (0-5 points).

SHARE Wave 8, being the last wave preceding the covid 19-pandemic, was set as the baseline for the cognitive function sum score. Word recall and serial sevens scores were summed, resulting in a general cognitive function score with a range of 0 – 25 (M: 13.79, SD: 3.92). The cut-off was defined at one standard deviation below the mean, and calculated separately for each country (range: 8 – 11), as shown in **Table S1**.

Table S1. Wave 8 number of participants, mean, standard deviation, and cut-off scores for cognitive function sum score per country.

Country	*N*	M (SD)	Cut-off
Austria	1 405	15.0 (3.87)	11
Belgium	1 789	14.5 (3.91)	11
Bulgaria	806	13.3 (3.52)	10
Croatia	1 024	13.3 (3.61)	10
Cyprus	411	12.0 (3.97)	8
Czech Republic	2 381	14.3 (3.49)	11
Denmark	1 948	14.8 (3.68)	11
Estonia	2 667	14.3 (4.15)	10
Finland	1 026	14.3 (3.71)	11
France	2 107	13.9 (3.95)	10
Germany	2 652	14.7 (3.61)	11
Greece	2 365	13.1 (3.54)	10
Hungary	715	14.1 (3.55)	11
Israel	667	13.6 (3.56)	10
Italy	1 702	12.2 (3.88)	8
Latvia	564	13.8 (3.72)	10
Lithuania	1 248	12.1 (3.90)	8
Luxembourg	818	15.2 (4.13)	11
Malta	653	12.0 (3.72)	8
Netherlands	1 805	14.2 (3.63)	11
Poland	1 702	12.5 (3.55)	9
Romania	1 072	12.5 (4.33)	8
Slovakia	944	14.0 (3.99)	10
Slovenia	2 165	13.5 (3.94)	10
Spain	1 383	11.7 (3.83)	8
Sweden	2 178	14.3 (3.72)	11
Switzerland	1 748	15.2 (3.88)	11

1.1.3 Longitudinal recall performance

In order to ensure that below-average cognitive performance was not transient at Wave 8, the single time point sum score was combined with longitudinal recall data. A similar approach has previously been used by Ahmadi-Abhari et al. (2017). With word recall data being the most readily available across SHARE waves (included in waves 1, 2, 4, 5, 6, 7 & 8), linear regression was used to calculate a slope for the sum of immediate and delayed recall scores (range: 0 – 20, m: 9.73, SD: 3.41), for each individual across all available time points (range: 2 – 7, 2: *N* = 9 915 [25.3 %], 3: *N* = 5 570 [14.2 %], 4: *N* = 8 417 [21.5 %], 5: *N* = 9 584 [24.4 %], 6: *N* = 2 793 [7.1 %], 7: *N* = 2 930 [7.5 %].

1.1.4 Classification of cognitive impairment

Taking both below-average performance and decline into account, cognitive impairment was defined as a cognitive function sum score below the country-specific cut-off at SHARE Wave 8, as well as a negative recall slope across time points. In the whole sample, 10.6 % were classified as cognitively impaired (*N* = 4 228, 2 218 females [52.5 %]). **Table S2** shows the percentage of participants being classified as cognitively impaired in different age groups. When cognitive function sum scores were plotted over time

Table S2. Number of participants and percentage of cognitive impairment in different age groups.

Age group (yrs)	50-59	60-69	70-79	80-89	90 +
**Total *N***	4 685	14 921	11 785	5 583	671
**Cognitively impaired (%)**	152 (3.24)	769 (5.15)	1 564 (11.72)	1 454 (26.04)	289 (43.07)

points, irrespective of SHARE Wave, the impaired and normal function groups deviated after the second time point, and the impaired group continued to decline while the normal group showed stable levels over time, as shown in **Figure S1**, panel A. Distributions of number of available time points for the cognitive sum score data were similar across groups (see **Figure S1**, panel B).

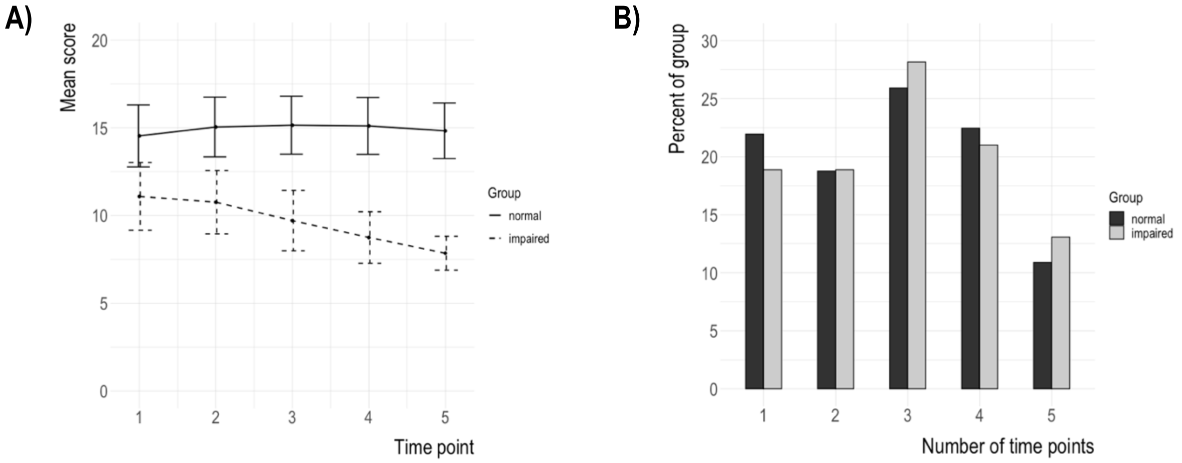

Figure S1. Longitudinal cognitive function sum scores and number of data time points in the normal and impaired cognitive function groups. **A)** Mean cognitive function sum score per time point and group. Error bars represent one standard deviation above and below the mean. **B)** Distribution of total number of time points as percentage of total group observations.

1.2 Validation analyses and results

In order to validate the cognitive impairment classification, the normal and impaired function groups were compared across a number of cognitive tests. In addition, a multiple logistic regression analysis was run using the cognitive impairment variable as outcome and known risk factors for age-related cognitive impairment as independent variables.

1.2.1 Group comparisons

Mean scores for the impaired and normal cognitive function groups were compared for five tests of cognitive function included in SHARE Wave 8 and not for computation of the cognitive function sum score: Verbal fluency, Orientation, Backwards count, Clock draw, and Visuospatial construction.

1.2.1.1 Test descriptions

The SHARE *Verbal fluency* test is a semantic fluency test, asking participants to name as many animals as they can within 60 seconds. The score refers to the number of unique correct responses

given within the time limit. The *Orientation* test contains four questions evaluating the participant's temporal orientation to date, month, year, and day of the week, with 1 point given for each correct answer. In *Backwards count*, participants are asked to count backwards from 20 as quickly as possible. Participants are given one opportunity to start over once if they want to. A score of 1 is given if the participant counts backwards once, from 20 to 11 or 19 to 10, without mistakes. If any mistake is made, a score of 0 is given. *Clock draw* is a cognitive screening test wherein participants are asked to draw a clock including all numbers and hands showing a specific time. A score of 1 represents correct drawing of clock face and hands. In *Visuospatial construction*, participants are asked to copy two designs: an infinity symbol and a three-dimensional cube. A score of 1 corresponds to one design correctly copied.

1.2.1.2 Results

The cognitive impairment group (N = 3 059, mean words: 15.1, SD = 6.12) performed significantly worse than the normal cognitive function group (N = 27 929, mean words: 21.5, SD = 7.37) on the Verbal fluency test $t(30\ 988) = 54.2$, $p > 0.001$, as well as on the other tests (results presented in **Table S3**).

Table S3. Results of Chi-Squared Tests comparing performance of the cognitively impaired and non-impaired groups on tests of cognitive function.

Variable	Value	Distribution of sample (%)		Number of observations		χ^2	*df*	*p*
		non-impaired	impaired	non-impaired	impaired			
Orientation	0	0.03	1.60	27 929	3 059	1407.9	4	< 0.001
	1	0.08	1.60					
	2	0.61	4.35					
	3	7.18	16.44					
	4	92.10	76.01					
Backwards count	0	1.95	9.07	19 503	2 425	407.4	1	< 0.001
	1	98.05	90.93					
Clock draw	0	17.34	38.21	17 012	4 119	541.4	1	< 0.001
	1	82.66	61.79					
Visuospatial construction	0	38.41	48.64	27 929	3 059	362.4	2	< 0.001
	1	16.80	24.29					
	2	44.78	27.07					

1.2.2 Weighted multiple logistic regression analysis

The relationship of cognitive impairment as outcome and several known risk factors for age-related cognitive dysfunction was investigated in a weighted multiple logistic regression analysis. Only Wave 8 data was used for the analysis (N = 30 099 after elimination of missing data [3.1 %], 17 056 females [56.67 %], mean age: 69.8 years, SD: 8.64, range: 52 – 103). The analysis was weighted using inverse probability weights according to each responder's probability of being part of SHARE Wave 8.

1.2.2.1 Independent variables

In addition to age (continuous), sex (male as reference), education level (less than upper secondary, and tertiary, with upper secondary as reference), having no cohabitant partner, engaging in no regular social or vigorous physical activities, BMI category (underweight and overweight, with normal as reference), having one or more IADL limitations, and scoring above the EURO-D depression scale cut off, all described in the main text, cardiovascular disease load and dementia were added as predictors. Cardiovascular load was treated as continuous and computed by summing responses to whether the respondent had ever been told by a doctor that they had a heart attack, hypertension, stroke, hyperglycaemia, or diabetes. Dementia was coded as 1 if the respondent reported ever been told by a doctor that they had dementia, and otherwise coded as 0. Country (with Germany as reference), and SHARE time point (continuous) were entered as control variables. Older age, lower education level, social isolation, physical inactivity, higher BMI, cardiovascular disease, depressive symptomatology, and IADL limitations have all been linked to cognitive impairment and dementia (Barberger-Gateau & Fabrigoule, 1997; Kivipelto et al., 2018; Livingston et al., 2020).

1.2.2.2 Results

Coefficients with standard errors, odds ratios with 95 % confidence interval, and significance levels are shown in **Table S4**. Older age, Male sex, less than upper secondary education, having no

Table S4. Logistic regression analysis results. Outcome: Cognitive impairment (1, N = 2 915; 0, N = 27 184). Covariates of no interest, not included in the table: Number of SHARE wave appearances & country of residence. Significance levels: ** = p < 0.01, *** = p <0.001.

**Variable**	coef.	se	OR	(95 % CI)	sig.
Intercept	-8.33	0.38	0.00	(0.00 - 0.00)	***
Age	0.08	0.01	1.08	(1.07 - 1.10)	***
Sex (female)	-0.41	0.09	0.67	(0.56 - 0.79)	***
Education level					
- *less than upper secondary*	0.56	0.10	1.76	(1.45 - 2.12)	***
- *tertiary*	-0.73	0.13	0.48	(0.38 - 0.62)	***
No cohabitant partner	0.25	0.09	1.28	(1.07 - 1.53)	**
No social activities	0.49	0.10	1.64	(1.34 - 2.01)	***
No vigorous physical activities	0.31	0.09	1.36	(1.15 - 1.61)	***
BMI					
- *overweight*	-0.03	0.08	0.97	(0.83 - 1.15)	
- *underweight*	-0.29	0.31	0.75	(0.41 - 1.36)	
IADL limitations	0.31	0.10	1.36	(1.13 - 1.64)	**
Cardiovascular load	-0.06	0.04	0.95	(0.88 - 1.02)	
EURO-D depression (≥ 4)	1.61	0.24	5.00	(3.10 - 8.07)	***
Dementia	0.34	0.09	1.40	(1.17 - 1.67)	***

cohabitant partner, engaging in no regular social or vigorous physical activities, having one or more IADL limitations, above cut-off depressive symptomatology, and reporting having been told by a doctor to have dementia were all significantly associated with a higher risk of being classified as cognitively impaired. Having tertiary education was associated with significantly less risk of cognitive impairment as compared to upper secondary education. The model had and 92 % of binned residuals falling within ± 2 standard deviations, and acceptable discriminative ability (AUC = 0.77).

1.3 Summary and conclusion

Employing a similar methodology to that used in another large survey (Ahmadi-Abhari et al., 2017), cognitive impairment was defined as below average cognitive function across three different cognitive function tests at one time point, in combination with declining word recall ability over at least two time points. Using this classification, we were able to identify individuals who performed worse on other cognitive function tests, and show that cognitive impairment as outcome was significantly associated with known risk factors for cognitive decline in older adults. We conclude that the adopted definition of cognitive impairment is valid in this sample.

2 Comparisons of loneliness group mean scores on the Three-Item Loneliness Scale

Table S5. Pairwise *t*-test comparisons of Three-Item Loneliness Scale mean scores of the no loneliness (1), situational loneliness (2), and persistent loneliness (3) groups at Survey of Health, Aging, and Retirement (SHARE) waves 5, 6, 7, and 8, displayed with Bonferroni-corrected *p*-values.

Wave	Group comparison			N 1	N 2	M 1	(SD 1)	M 2	(SD 2)	*p*
5	No loneliness	-	Situational	13 080	2 006	3.49	(0.97)	3.80	(1.19)	< 0.0001
5	No loneliness	-	Persistent	13 080	2 127	3.49	(0.97)	4.57	(1.65)	< 0.0001
5	Situational	-	Persistent	2 006	2 127	3.80	(1.19)	4.57	(1.65)	< 0.0001
6	No loneliness	-	Situational	15 877	2 619	3.59	(1.02)	3.99	(1.25)	< 0.0001
6	No loneliness	-	Persistent	15 877	2 991	3.59	(1.02)	4.97	(1.70)	< 0.0001
6	Situational	-	Persistent	2 619	2 991	3.99	(1.25)	4.97	(1.70)	< 0.0001
7	No loneliness	-	Situational	3 954	665	3.54	(0.98)	3.89	(1.14)	< 0.0001
7	No loneliness	-	Persistent	3 954	814	3.54	(0.98)	5.08	(1.72)	< 0.0001
7	Situational	-	Persistent	665	814	3.89	(1.14)	5.08	(1.72)	< 0.0001
8	No loneliness	-	Situational	22 311	3 675	3.58	(1.03)	3.70	(0.98)	< 0.0001
8	No loneliness	-	Persistent	22 311	4 190	3.58	(1.03)	5.61	(1.64)	< 0.0001
8	Situational	-	Persistent	3 675	4 190	3.70	(0.98)	5.61	(1.64)	< 0.0001

3 Predictors for situational over no loneliness, persistent over no loneliness, and persistent over situational loneliness

Table S6. Analysis 1, outcome: Situational loneliness (1) – No loneliness (0).

Block	Variable	Model 1<br>coef. se OR (95 % CI) sig.	Model 2<br>coef. se OR (95 % CI) sig.	Model 3<br>coef. se OR (95 % CI) sig.	Model 4<br>coef. se OR (95 % CI) sig.	Model 5<br>coef. se OR (95 % CI) sig.
	Intercept	**-2.48 0.29 0.08 (0.05 - 0.15) *****	**-3.45 0.47 0.03 (0.01 - 0.08) *****	**-3.27 0.51 0.04 (0.01 - 0.10) *****	**-2.95 0.58 0.05 (0.02 - 0.16) *****	**-2.85 0.54 0.06 (0.02 - 0.17) *****
1 Geographic region	Northern Europe	-0.15 0.11 0.86 (0.70 - 1.06)	-0.21 0.11 0.81 (0.66 - 1.00)	-0.20 0.11 0.82 (0.67 - 1.01)	-0.18 0.11 0.84 (0.68 - 1.03)	**-0.27 0.14 0.76 (0.58 - 1.00) ***
	Southern Europe	**0.46 0.15 1.58 (1.18 - 2.11) ****	**0.45 0.14 1.58 (1.21 - 2.05) *****	**0.46 0.14 1.58 (1.20 - 2.07) *****	**0.50 0.14 1.65 (1.26 - 2.15) *****	0.21 0.21 1.23 (0.82 - 1.84)
	Eastern Europe	**0.38 0.12 1.47 (1.17 - 1.85) ****	**0.32 0.12 1.38 (1.09 - 1.74) ****	**0.28 0.13 1.32 (1.03 - 1.69) ***	**0.29 0.13 1.33 (1.04 - 1.71) ***	0.23 0.13 1.26 (0.98 - 1.63)
	Israel	0.42 0.43 1.53 (0.65 - 3.57)	0.23 0.51 1.26 (0.47 - 3.41)	0.26 0.50 1.30 (0.49 - 3.44)	0.23 0.48 1.26 (0.50 - 3.21)	-0.16 0.46 0.85 (0.35 - 2.08)
2 Demographic variables	Age		**0.01 0.01 1.01 (1.00 - 1.02) ***	0.01 0.01 1.01 (0.99 - 1.02)	0.00 0.01 1.00 (0.99 - 1.01)	0.00 0.01 1.00 (0.98 - 1.01)
	Sex (female)		**0.64 0.10 1.90 (1.57 - 2.31) *****	**0.54 0.10 1.72 (1.41 - 2.10) *****	**0.47 0.11 1.60 (1.30 - 1.98) *****	**0.46 0.10 1.58 (1.29 - 1.93) *****
	Education level					
	- less than upper secondary		0.17 0.12 1.18 (0.94 - 1.49)	0.17 0.12 1.19 (0.95 - 1.49)	0.15 0.12 1.16 (0.93 - 1.46)	0.15 0.10 1.16 (0.95 - 1.43)
	- tertiary		0.09 0.12 1.09 (0.87 - 1.37)	0.12 0.11 1.13 (0.90 - 1.41)	0.14 0.11 1.15 (0.92 - 1.43)	0.14 0.11 1.14 (0.92 - 1.43)
	Migrant		0.06 0.20 1.06 (0.71 - 1.58)	0.03 0.21 1.03 (0.68 - 1.57)	0.00 0.21 1.00 (0.66 - 1.51)	0.00 0.21 1.00 (0.66 - 1.52)
	Nursing home		-0.27 0.40 0.76 (0.35 - 1.66)	-0.53 0.40 0.59 (0.27 - 1.28)	-0.57 0.41 0.57 (0.25 - 1.25)	-0.72 0.41 0.49 (0.22 - 1.10)
	Area of residence					
	- large town/suburb		-0.16 0.21 0.86 (0.56 - 1.30)	-0.14 0.22 0.87 (0.57 - 1.34)	-0.14 0.22 0.87 (0.57 - 1.33)	-0.16 0.20 0.86 (0.58 - 1.26)
	- small town/village		-0.29 0.21 0.75 (0.50 - 1.12)	-0.27 0.21 0.77 (0.50 - 1.16)	-0.26 0.21 0.77 (0.50 - 1.17)	-0.28 0.19 0.76 (0.52 - 1.09)
3 Objective and subjective social network variables	No cohabitant partner			**0.45 0.13 1.57 (1.22 - 2.00) *****	**0.44 0.13 1.55 (1.20 - 1.98) *****	**0.47 0.13 1.60 (1.25 - 2.05) *****
	Widowed			0.18 0.13 1.19 (0.92 - 1.55)	0.17 0.14 1.19 (0.91 - 1.55)	0.17 0.13 1.18 (0.91 - 1.54)
	No children			0.03 0.16 1.03 (0.75 - 1.41)	0.01 0.16 1.01 (0.74 - 1.39)	-0.02 0.16 0.98 (0.71 - 1.34)
	Network size					
	- small network (< 2)			-0.02 0.12 0.98 (0.78 - 1.24)	0.01 0.12 1.01 (0.80 - 1.27)	-0.01 0.11 0.99 (0.79 - 1.23)
	- large network (> 3)			0.00 0.10 1.00 (0.82 - 1.22)	-0.01 0.10 0.99 (0.82 - 1.20)	0.02 0.10 1.02 (0.84 - 1.23)
	No social activities			0.20 0.11 1.22 (0.99 - 1.50)	0.17 0.10 1.18 (0.96 - 1.45)	0.16 0.10 1.18 (0.96 - 1.44)
	Low network satisfaction			0.10 0.13 1.10 (0.85 - 1.43)	0.08 0.13 1.08 (0.84 - 1.40)	0.08 0.13 1.08 (0.84 - 1.39)
4 Physical & cognitive health variables	Two or more chronic diseases				**0.24 0.09 1.27 (1.06 - 1.52) ***	**0.25 0.09 1.28 (1.07 - 1.54) ****
	IADL limitations				0.12 0.10 1.12 (0.92 - 1.37)	0.09 0.10 1.09 (0.89 - 1.34)
	BMI					
	- overweight				-0.11 0.11 0.89 (0.72 - 1.12)	-0.11 0.11 0.90 (0.72 - 1.12)
	- underweight				0.22 0.61 1.25 (0.38 - 4.13)	0.23 0.63 1.26 (0.36 - 4.33)
	No vigorous physical activities				-0.03 0.11 0.97 (0.79 - 1.19)	-0.05 0.09 0.95 (0.79 - 1.14)
	Alcohol				**-0.54 0.17 0.58 (0.41 - 0.82) ****	**-0.60 0.18 0.55 (0.39 - 0.78) *****
	Cognitive impairment				0.06 0.12 1.07 (0.84 - 1.36)	0.08 0.12 1.08 (0.85 - 1.37)
	EURO-D depression (≥ 4)				**0.29 0.09 1.34 (1.12 - 1.60) ****	**0.28 0.09 1.33 (1.10 - 1.59) ****
5 Pandemic variables	Deaths per million					0.00 0.00 1.00 (1.00 - 1.00)
	Older pop. isolation months^2					0.02 0.03 1.02 (0.96 - 1.08)
	Older pop. isolation months					-0.01 0.13 0.99 (0.77 - 1.28)
	Stayed home since outbreak					**0.42 0.21 1.53 (1.01 - 2.31) ***
	Seldom personal contact					**0.30 0.10 1.35 (1.11 - 1.65) ****

Significance levels: * = $p < 0.05$, ** = $p < 0.01$, *** = $p < 0.001$. Bold characters indicate statistical significance in the model.

Table S7. Analysis 2, outcome: Persistent loneliness (1) – No loneliness (0).

Block	Variable	Model 1					Model 2					Model 3					Model 4					Model 5				
		coef.	se	OR	(95 % CI)	sig.	coef.	se	OR	(95 % CI)	sig.	coef.	se	OR	(95 % CI)	sig.	coef.	se	OR	(95 % CI)	sig.	coef.	se	OR	(95 % CI)	sig.
	Intercept	**-2.28**	**0.20**	**0.10**	**(0.07 - 0.15)**	***	**-3.27**	**0.38**	**0.04**	**(0.02 - 0.08)**	***	**-4.07**	**0.41**	**0.02**	**(0.01 - 0.04)**	***	**-3.46**	**0.45**	**0.03**	**(0.01 - 0.08)**	***	**-4.33**	**0.48**	**0.01**	**(0.01 - 0.03)**	***
1 Geographic region	Northern Europe	-0.13	0.11	0.87	(0.70 - 1.09)		-0.17	0.12	0.84	(0.67 - 1.06)		-0.09	0.12	0.91	(0.72 - 1.16)		0.01	0.12	1.01	(0.80 - 1.27)		**-0.31**	**0.13**	**0.73**	**(0.56 - 0.95)**	*
	Southern Europe	**0.39**	**0.10**	**1.47**	**(1.20 - 1.80)**	***	**0.28**	**0.10**	**1.32**	**(1.08 - 1.62)**	**	**0.35**	**0.12**	**1.43**	**(1.13 - 1.79)**	**	**0.43**	**0.12**	**1.53**	**(1.20 - 1.96)**	***	-0.23	0.20	0.79	(0.53 - 1.18)	
	Eastern Europe	**0.46**	**0.11**	**1.58**	**(1.26 - 1.97)**	***	**0.34**	**0.11**	**1.40**	**(1.13 - 1.75)**	**	**0.30**	**0.13**	**1.35**	**(1.05 - 1.74)**	*	**0.29**	**0.14**	**1.34**	**(1.03 - 1.75)**	*	**0.28**	**0.14**	**1.33**	**(1.01 - 1.75)**	*
	Israel	0.30	0.40	1.35	(0.62 - 2.93)		0.02	0.38	1.02	(0.48 - 2.16)		0.07	0.35	1.07	(0.53 - 2.15)		0.05	0.42	1.05	(0.46 - 2.38)		**-0.95**	**0.47**	**0.39**	**(0.15 - 0.97)**	*
2 Demographic variables	Age						**0.03**	**0.00**	**1.03**	**(1.02 - 1.04)**	***	**0.01**	**0.00**	**1.01**	**(1.00 - 1.02)**	*	0.00	0.01	1.00	(0.99 - 1.01)		0.00	0.01	1.00	(0.99 - 1.01)	
	Sex (female)						**0.76**	**0.08**	**2.13**	**(1.82 - 2.49)**	***	**0.46**	**0.09**	**1.59**	**(1.33 - 1.90)**	***	**0.26**	**0.09**	**1.29**	**(1.08 - 1.56)**	**	**0.24**	**0.09**	**1.27**	**(1.06 - 1.53)**	*
	Education level																									
	- less than upper secondary						**0.41**	**0.09**	**1.50**	**(1.26 - 1.79)**	***	**0.42**	**0.10**	**1.52**	**(1.25 - 1.84)**	***	**0.31**	**0.10**	**1.37**	**(1.12 - 1.67)**	**	**0.34**	**0.11**	**1.41**	**(1.14 - 1.73)**	**
	- tertiary						-0.06	0.10	0.95	(0.78 - 1.14)		0.06	0.11	1.06	(0.86 - 1.31)		0.17	0.11	1.19	(0.96 - 1.47)		0.20	0.11	1.22	(0.99 - 1.52)	
	Migrant						0.26	0.15	1.30	(0.97 - 1.72)		0.19	0.16	1.21	(0.88 - 1.67)		0.09	0.17	1.10	(0.79 - 1.52)		0.08	0.16	1.09	(0.79 - 1.50)	
	Nursing home						0.07	0.33	1.07	(0.56 - 2.06)		-0.43	0.33	0.65	(0.34 - 1.25)		**-0.70**	**0.32**	**0.50**	**(0.27 - 0.93)**	*	**-0.75**	**0.32**	**0.47**	**(0.25 - 0.89)**	*
	Area of residence																									
	- large town/suburb						-0.15	0.12	0.86	(0.68 - 1.08)		-0.09	0.13	0.92	(0.72 - 1.17)		-0.13	0.13	0.87	(0.68 - 1.13)		-0.16	0.13	0.85	(0.66 - 1.10)	
	- small town/village						**-0.31**	**0.10**	**0.74**	**(0.60 - 0.91)**	**	-0.19	0.11	0.82	(0.66 - 1.03)		-0.23	0.12	0.80	(0.63 - 1.00)		**-0.25**	**0.12**	**0.78**	**(0.62 - 0.98)**	*
3 Objective and subjective social network variables	No cohabitant partner											**1.47**	**0.11**	**4.33**	**(3.48 - 5.39)**	***	**1.40**	**0.11**	**4.07**	**(3.26 - 5.09)**	***	**1.44**	**0.11**	**4.21**	**(3.36 - 5.27)**	***
	Widowed											**0.36**	**0.11**	**1.44**	**(1.16 - 1.78)**	***	**0.39**	**0.11**	**1.48**	**(1.18 - 1.85)**	***	**0.38**	**0.12**	**1.47**	**(1.17 - 1.84)**	***
	No children											0.10	0.14	1.11	(0.85 - 1.45)		0.13	0.14	1.13	(0.86 - 1.50)		0.10	0.14	1.11	(0.83 - 1.47)	
	Network size																									
	- small network (< 2)											0.05	0.10	1.05	(0.87 - 1.27)		0.06	0.10	1.06	(0.87 - 1.30)		0.04	0.10	1.04	(0.85 - 1.28)	
	- large network (> 3)											-0.18	0.10	0.83	(0.69 - 1.01)		-0.18	0.10	0.83	(0.69 - 1.01)		-0.16	0.10	0.86	(0.70 - 1.04)	
	No social activities											0.15	0.10	1.16	(0.96 - 1.41)		-0.04	0.10	0.96	(0.78 - 1.17)		-0.05	0.10	0.95	(0.77 - 1.16)	
	Low network satisfaction											**0.95**	**0.12**	**2.57**	**(2.02 - 3.28)**	***	**0.81**	**0.13**	**2.25**	**(1.76 - 2.88)**	***	**0.80**	**0.13**	**2.22**	**(1.73 - 2.84)**	***
4 Physical & cognitive health variables	Two or more chronic diseases																0.17	0.09	1.19	(0.99 - 1.43)		**0.19**	**0.09**	**1.21**	**(1.01 - 1.46)**	*
	IADL limitations																**0.32**	**0.11**	**1.38**	**(1.11 - 1.72)**	**	**0.30**	**0.11**	**1.35**	**(1.09 - 1.69)**	**
	BMI																									
	- overweight																-0.09	0.09	0.92	(0.77 - 1.10)		-0.09	0.09	0.91	(0.76 - 1.10)	
	- underweight																-0.19	0.26	0.82	(0.50 - 1.37)		-0.20	0.26	0.82	(0.50 - 1.36)	
	No vigorous physical activities																0.11	0.09	1.11	(0.93 - 1.34)		0.12	0.09	1.13	(0.94 - 1.36)	
	Alcohol																-0.09	0.25	0.92	(0.56 - 1.49)		-0.12	0.25	0.89	(0.54 - 1.45)	
	Cognitive impairment																0.17	0.12	1.19	(0.95 - 1.50)		0.17	0.12	1.19	(0.95 - 1.50)	
	EURO-D depression (≥ 4)																**1.26**	**0.09**	**3.54**	**(2.98 - 4.20)**	***	**1.27**	**0.09**	**3.55**	**(2.99 - 4.22)**	***
5 Pandemic variables	Deaths per million																					**-0.00**	**0.00**	**1.00**	**(1.00 - 1.00)**	**
	Older pop. isolation months^2																					-0.05	0.03	0.95	(0.90 - 1.00)	
	Older pop. isolation months																					**0.69**	**0.14**	**2.00**	**(1.51 - 2.66)**	***
	Stayed home since outbreak																					0.15	0.11	1.17	(0.94 - 1.45)	
	Seldom personal contact																					**0.17**	**0.09**	**1.19**	**(1.00 - 1.41)**	*

Significance levels: * = $p < 0.05$, ** = $p < 0.01$, *** = $p < 0.001$. Bold characters indicate statistical significance in the model.

Table S8. Analysis 3, outcome: Persistent loneliness (1) – Situational loneliness (0).

Block	Variable	Model 1					Model 2					Model 3					Model 4					Model 5				
		coef.	se	OR	(95 % CI)	sig.	coef.	se	OR	(95 % CI)	sig.	coef.	se	OR	(95 % CI)	sig.	coef.	se	OR	(95 % CI)	sig.	coef.	se	OR	(95 % CI)	sig.
	Intercept	**0.21**	**0.31**	**1.23**	**(0.67 - 2.27)**	*	-1.02	0.51	0.36	(0.13 - 0.99)		-0.92	0.56	0.40	(0.13 - 1.19)		-0.77	0.58	0.46	(0.15 - 1.45)		-1.10	0.62	0.33	(0.10 - 1.11)	
1 Geographic region	Northern Europe	0.02	0.14	1.02	(0.77 - 1.36)		-0.01	0.15	0.99	(0.74 - 1.33)		0.01	0.15	1.01	(0.75 - 1.37)		0.12	0.15	1.13	(0.84 - 1.52)		-0.09	0.17	0.91	(0.65 - 1.28)	
	Southern Europe	-0.08	0.16	0.92	(0.67 - 1.27)		-0.18	0.15	0.84	(0.62 - 1.14)		0.00	0.16	1.00	(0.72 - 1.38)		0.21	0.16	1.23	(0.90 - 1.68)		-0.22	0.26	0.80	(0.48 - 1.34)	
	Eastern Europe	0.06	0.15	1.06	(0.79 - 1.43)		0.01	0.15	1.01	(0.75 - 1.36)		-0.02	0.16	0.98	(0.71 - 1.36)		0.04	0.16	1.04	(0.75 - 1.43)		0.09	0.17	1.10	(0.79 - 1.53)	
	Israel	-0.22	0.54	0.81	(0.28 - 2.33)		-0.29	0.57	0.75	(0.24 - 2.32)		-0.49	0.61	0.62	(0.18 - 2.05)		-0.53	0.79	0.59	(0.13 - 2.79)		-0.97	0.77	0.38	(0.08 - 1.72)	
2 Demographic variables	Age						**0.02**	**0.01**	**1.02**	**(1.01 - 1.03)**	**	0.00	0.01	1.00	(0.99 - 1.02)		0.00	0.01	1.00	(0.98 - 1.01)		0.00	0.01	1.00	(0.98 - 1.01)	
	Sex (female)						0.15	0.11	1.16	(0.93 - 1.45)		0.05	0.12	1.05	(0.83 - 1.32)		-0.08	0.12	0.92	(0.73 - 1.16)		-0.10	0.11	0.91	(0.72 - 1.13)	
	Education level																									
	- less than upper secondary						**0.26**	**0.13**	**1.30**	**(1.02 - 1.66)**	*	0.24	0.12	1.27	(1.00 - 1.61)		0.11	0.12	1.12	(0.88 - 1.42)		0.14	0.12	1.15	(0.90 - 1.45)	
	- tertiary						-0.14	0.14	0.87	(0.66 - 1.14)		-0.16	0.15	0.85	(0.63 - 1.14)		-0.01	0.14	0.99	(0.75 - 1.32)		-0.01	0.15	0.99	(0.74 - 1.32)	
	Migrant						0.21	0.22	1.23	(0.81 - 1.89)		0.29	0.21	1.34	(0.89 - 2.02)		0.26	0.21	1.30	(0.87 - 1.95)		0.27	0.20	1.31	(0.88 - 1.95)	
	Nursing home						0.47	0.40	1.60	(0.73 - 3.53)		0.20	0.45	1.22	(0.50 - 2.96)		-0.12	0.39	0.89	(0.41 - 1.92)		-0.07	0.40	0.93	(0.43 - 2.04)	
	Area of residence																									
	- large town/suburb						0.04	0.21	1.04	(0.69 - 1.57)		0.08	0.20	1.09	(0.73 - 1.61)		0.10	0.19	1.10	(0.76 - 1.61)		0.05	0.18	1.05	(0.73 - 1.50)	
	- small town/village						-0.03	0.19	0.97	(0.67 - 1.41)		0.06	0.18	1.06	(0.75 - 1.50)		0.04	0.17	1.04	(0.75 - 1.46)		-0.01	0.16	0.99	(0.72 - 1.36)	
3 Objective and subjective social network variables	No cohabitant partner											**1.05**	**0.15**	**2.86**	**(2.13 - 3.84)**	***	**1.07**	**0.15**	**2.91**	**(2.16 - 3.92)**	***	**1.05**	**0.15**	**2.87**	**(2.13 - 3.87)**	***
	Widowed											0.13	0.15	1.14	(0.85 - 1.52)		0.19	0.15	1.21	(0.90 - 1.62)		0.22	0.15	1.24	(0.93 - 1.67)	
	No children											0.13	0.19	1.14	(0.78 - 1.65)		0.19	0.18	1.21	(0.84 - 1.73)		0.19	0.19	1.21	(0.84 - 1.74)	
	Network size																									
	- small network (< 2)											0.13	0.14	1.14	(0.87 - 1.49)		0.13	0.13	1.14	(0.87 - 1.48)		0.13	0.13	1.13	(0.87 - 1.48)	
	- large network (> 3)											-0.19	0.12	0.83	(0.65 - 1.05)		-0.21	0.12	0.81	(0.64 - 1.02)		-0.21	0.12	0.81	(0.64 - 1.03)	
	No social activities											-0.03	0.14	0.97	(0.74 - 1.27)		-0.18	0.13	0.83	(0.65 - 1.07)		-0.19	0.13	0.83	(0.64 - 1.07)	
	Low network satisfaction											**0.86**	**0.15**	**2.37**	**(1.78 - 3.16)**	***	**0.72**	**0.15**	**2.06**	**(1.54 - 2.75)**	***	**0.71**	**0.15**	**2.04**	**(1.53 - 2.74)**	***
4 Physical & cognitive health variables	Two or more chronic diseases																0.05	0.11	1.05	(0.85 - 1.30)		0.07	0.11	1.07	(0.86 - 1.33)	
	IADL limitations																**0.31**	**0.12**	**1.36**	**(1.08 - 1.73)**	*	**0.34**	**0.12**	**1.40**	**(1.10 - 1.78)**	**
	BMI																									
	- overweight																-0.03	0.11	0.97	(0.77 - 1.21)		-0.05	0.11	0.95	(0.76 - 1.19)	
	- underweight																-0.86	0.67	0.42	(0.11 - 1.58)		-0.92	0.71	0.40	(0.10 - 1.61)	
	No vigorous physical activities																0.13	0.11	1.14	(0.91 - 1.41)		0.16	0.11	1.17	(0.95 - 1.45)	
	Alcohol																**0.68**	**0.31**	**1.97**	**(1.07 - 3.63)**	*	**0.67**	**0.30**	**1.96**	**(1.08 - 3.56)**	*
	Cognitive impairment																0.17	0.15	1.19	(0.89 - 1.59)		0.19	0.15	1.21	(0.90 - 1.62)	
	EURO-D depression (≥ 4)																**1.04**	**0.10**	**2.82**	**(2.30 - 3.47)**	***	**1.04**	**0.10**	**2.83**	**(2.30 - 3.47)**	***
5 Pandemic variables	Deaths per million																					0.00	0.00	1.00	(1.00 - 1.00)	
	Older pop. isolation months																					**0.21**	**0.09**	**1.24**	**(1.03 - 1.48)**	*
	Stayed home since outbreak																					-0.22	0.15	0.80	(0.60 - 1.08)	
	Seldom personal contact																					-0.09	0.11	0.92	(0.74 - 1.14)	

Significance levels: * = $p < 0.05$, ** = $p < 0.01$, *** = $p < 0.001$. Bold characters indicate statistical significance in the model.

4 References

Ahmadi-Abhari, S., Guzman-Castillo, M., Bandosz, P., Shipley, M. J., Muniz-Terrera, G., Singh-Manoux, A., et al. (2017). Temporal trend in dementia incidence since 2002 and projections for prevalence in England and Wales to 2040: Modelling study. *BMJ (Online)* 358. doi: 10.1136/bmj.j2856.

Barberger-Gateau, P., and Fabrigoule, C. (1997). Disability and cognitive impairment in the elderly. *Disabil Rehabil* 19, 175–193. doi: 10.3109/09638289709166525.

Crimmins, E. M., Kim, J. K., Langa, K. M., and Weir, D. R. (2011). Assessment of cognition using surveys and neuropsychological assessment: the Health and Retirement Study and the Aging, Demographics, and Memory Study. *J Gerontol B Psychol Sci Soc Sci* 66 Suppl 1. doi: 10.1093/geronb/gbr048.

Kivipelto, M., Mangialasche, F., and Ngandu, T. (2018). Lifestyle interventions to prevent cognitive impairment, dementia and Alzheimer disease. *Nat Rev Neurol* 14, 653–666. doi: 10.1038/s41582-018-0070-3.

Langa, K. M. (2020). Langa-Weir classification of cognitive function (1995 Onward). *Survey Research Center Institute for Social Research, University of Michigan*.

Livingston, G., Huntley, J., Sommerlad, A., Ames, D., Ballard, C., Banerjee, S., et al. (2020). Dementia prevention, intervention, and care: 2020 report of the Lancet Commission. *The Lancet* 396, 413–446. doi: 10.1016/S0140-6736(20)30367-6.
